# Candida Shunt Infection Causing Arachnoiditis and Hydrocephalus: A Case Report

**DOI:** 10.7759/cureus.23675

**Published:** 2022-03-31

**Authors:** Jason J Kim, Chenxuan Li, Simon G Ammanuel, Ahmed M Elbayomy, Paul S Page, Azam S Ahmed

**Affiliations:** 1 Neurological Surgery, University of Wisconsin School of Medicine and Public Health, Madison, USA; 2 Neurological Surgery, University of Wisconsin Hospital and Clinics, Madison, USA

**Keywords:** fungal infection, hydrocephalus, arachnoiditis, vp shunt, candida

## Abstract

Arachnoiditis is a relatively rare condition and can result in long-term chronic and debilitating complications if not diagnosed early and treated properly. However, diagnosis of arachnoiditis is rare and knowledge of potential causes of this condition is still sparse. Current known causes of arachnoiditis include infections, trauma, spinal tumors, and iatrogenic causes induced via neurological interventions.

Here, we present a case of a 65-year-old female who presented with arachnoiditis caused by *Candida albicans* infection from a contaminated ventriculoperitoneal (VP) shunt, placed following the development of hydrocephalus from subarachnoid hemorrhage. During her initial assessment, the possibility of arachnoiditis was raised after spinal magnetic resonance imaging (MRI) due to leg weakness and spasms with bladder dysfunction. However, further workup was not pursued after a normal spinal angiogram and lack of constitutional symptoms. She presented six months later with symptoms of fever and lower abdominal pain. She was diagnosed with fungal arachnoiditis after a computerized tomography (CT) of the abdomen showed thickening of the fascia around the shunt catheter and fluid collections near the tip of the shunt in the abdominal cavity after hospitalization. The diagnosis was made after an ultrasound-guided tap of the same area revealed budding yeast and cerebrospinal fluid (CSF) showed growths of *Candida albicans*. Her shunt was removed, and she received intravenous (IV) antifungals and recovered.

MRI should be considered with clinical presentations that are characteristic of arachnoiditis. Symptoms from fungal infections are usually dramatic; however, in some instances as in this case, they may follow a more progressive course. The patient should be extensively evaluated for infection, especially fungal, in interventions involving device placement even when minimally, but persistently, symptomatic.

## Introduction

Arachnoiditis is a rare disease that can lead to long-term disability [1-7]. It involves inflammation of the spinal cord and nerve roots due to fibrosis and adhesion of the arachnoid [1-3,7,8]. It can be asymptomatic in some instances, while the symptoms can also vary from mild pain or numbness to paraplegia [3,7,9]. The etiology includes primary infections, trauma, spinal tumors, subarachnoid hemorrhage, and iatrogenic causes such as neurosurgical interventions [3,5,7]. We present in this paper a case of arachnoiditis caused by candida shunt infection, which led to hydrocephalus and replacement of the original shunt.

## Case presentation

Initial evaluation

**Figure 1:**
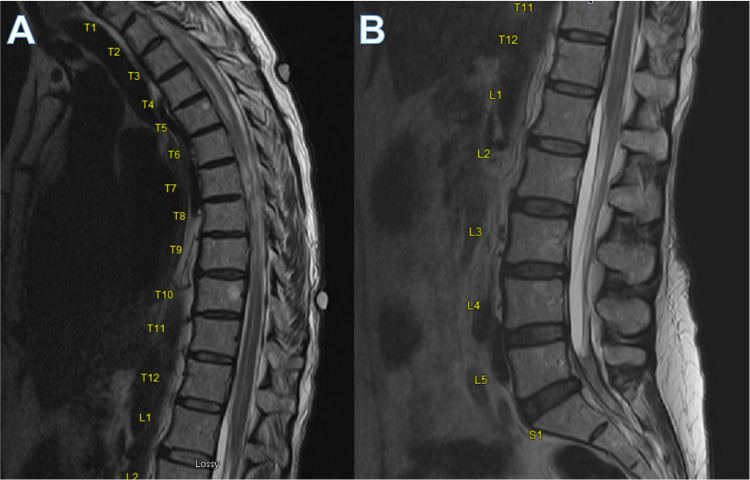
MRI thoracic (A) and lumbar spine (B) demonstrating variability in the signal of the spinal fluid at different levels with possible arachnoid webs and septation. The levels are noted next to each spinal column. The arrows denote MRI findings of possible adherence of the cord to the periphery with nerve conglomeration, which is consistent with group 1 and 2 findings of arachnoiditis.

The patient is a 65-year-old female who previously had a right posterior communicating artery aneurysmal subarachnoid hemorrhage treated with coil embolization in June 2020. Her recovery was complicated by hydrocephalus one month after her presentation requiring VP shunt placement at an outside facility. There was significant coil compaction within two months of embolization for which she underwent a flow diverter placement in September 2020. In October of the same year, she was briefly admitted with confusion, which was resolved with observation. She developed low back pain that was radiating to the buttock and posterior thigh and calf regions bilaterally. Following the resolution of her back pain, she developed thoracic muscle spasms similar to her chronic muscle spasms but more severe. She also described episodic leg weakness and urinary retention. Spinal imaging in September and November was notable for variability in the spinal fluid signal at different levels with possible arachnoid webs and septation (Figure 1).

A differential diagnosis of arachnoiditis was raised. Fungal infection was considered but deemed less likely due to the absence of constitutional symptoms and only moderate, non-disabling pain. Other bacterial etiologies were considered but deemed less likely given lack of abscess on imaging, symptoms notable for meningitis, and constitutional symptoms of infection. A lumbar puncture was notable for exceedingly slow flow with the retrieval of only 3 mL of CSF after 30 minutes. The fluid analysis demonstrated a lymphocytic pleocytosis and a protein level greater than 2000 mg/dl. The cerebrospinal fluid beta-glucan was positive. She had negative cryptococcal antigen and coccidiosis antigen. Blastomycosis and histoplasmosis analysis was negative. Serum neuromyelitis optical (NMO) and myelin oligodendrocyte glycoprotein (MOG) antibodies were negative. A spinal angiogram was normal. Additional opinions were obtained outside our institution which were unfruitful. 

Hospital course

She ultimately presented to an outside emergency department with fever and lower-non-radiating abdominal pain in April 2021 (eight months post VP shunt placement). At the outside hospital, she had a CT abdomen and pelvis that showed mesenteric stranding and fascial thickening around the distal portion of the VP shunt catheter. Piperacillin and tazobactam and doxycycline were started but later received tigecycline and meropenem, leading to transfer to our hospital for further evaluation. Due to a lack of outside hospital records, access to doses of the antibiotic regimen could not be obtained. However, bacterial and fungal cultures were reported to be negative by the outside hospital provider. During our hospitalization, she initially received 1.25 grams of IV vancomycin every eight hours, 2g IV cefepime every eight hours, and 500 mg of oral metronidazole every eight hours, which improved her mental status although she remained encephalopathic. Initially CSF fungal testing and interferon-gamma release assay (IGRA) were both unremarkable.

**Figure 2:**
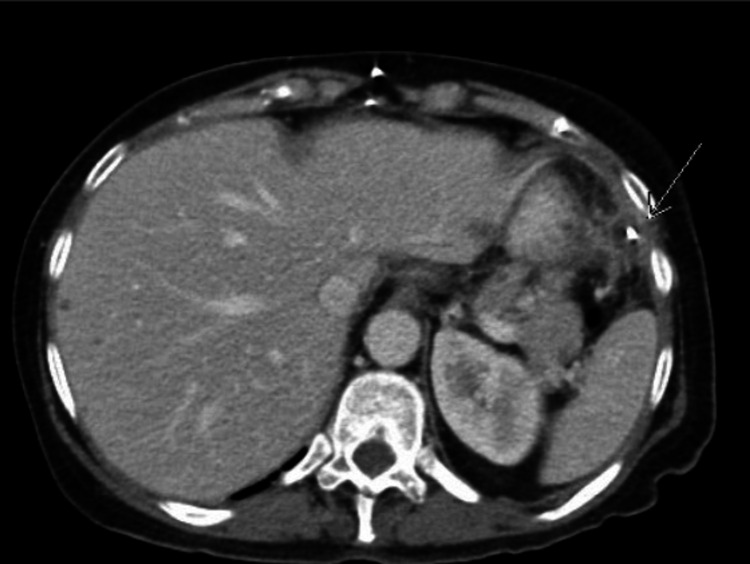
CT abdomen and pelvis with contrast showing fluid collection hypodense around the VP shunt denoted with a white arrow.

**Figure 3:**
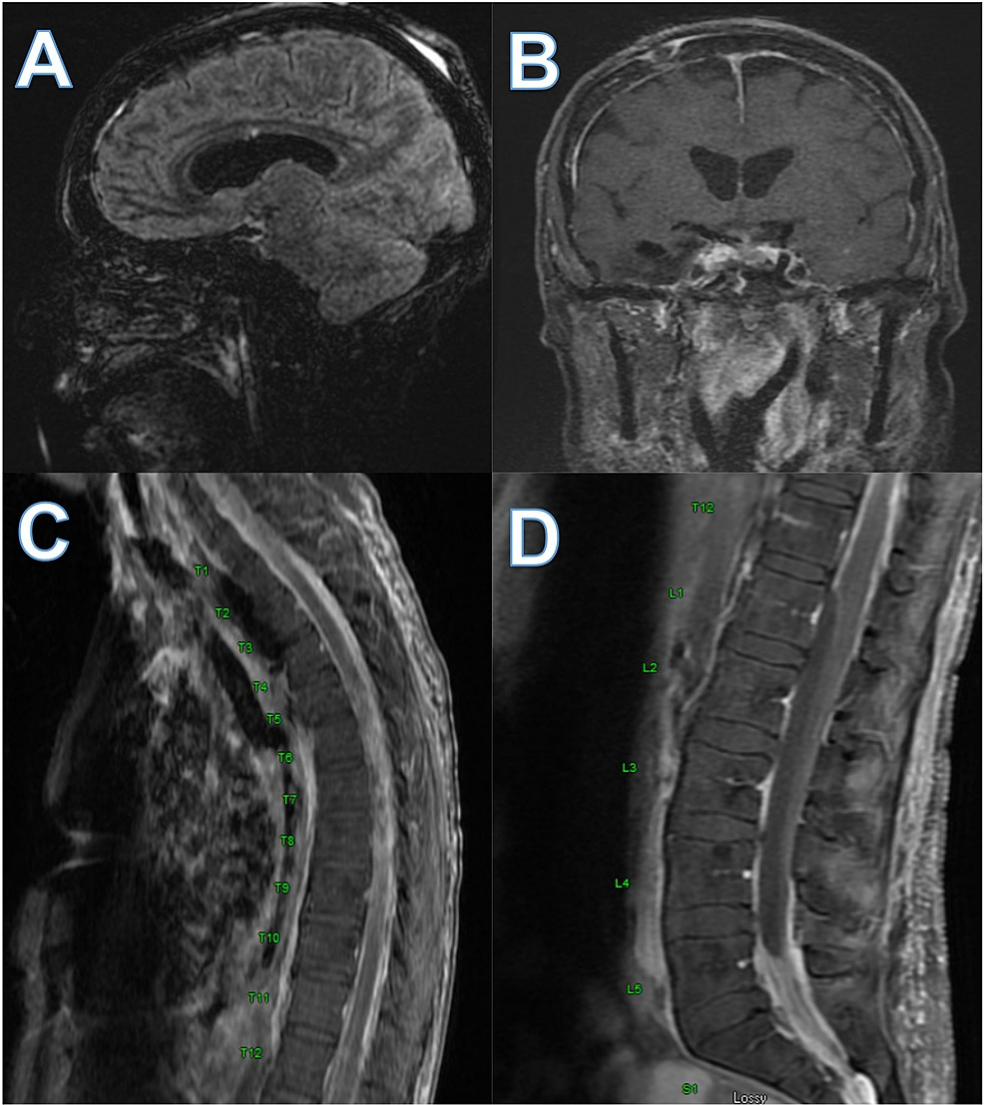
Head MRI and MRI total spine showing chronic adhesive arachnoiditis MRI T2 flair of sagittal (A) and coronal (B) sections demonstrating T2 intensity at the basilar cisterns denoted by the arrows and MRI T2 thoracic (C) and lumbar (D) spine also showing T2 intensity, adhesion, and hypodense portion of lumbar spinal cord consistent with group 2 and group 3 arachnoiditis.

One week into the hospital stay, she had worsening of her altered mental status and was intubated with stat head CT, which was normal. She was moved to the Neurological ICU and found to have status epilepticus. A repeat CT abdomen pelvis showed small fluid collections adjacent to the tip of the VP catheter shunt (Figure 2). An ultrasound-guided tap of the distal abdominal catheter area revealed budding yeast-like cells with pseudohyphae. The shunt was externalized and clamped, and micafungin was added. CSF cultures of distal shunt also revealed budding yeast and pseudohyphae, which was *Candida albicans*. Total removal of VP shunt was performed with external ventricular drain (EVD) placement with no biofilm observed during removal. CSF was sent at the time of the procedure, which grew *Candida* *albicans.* She was started on 5 milligrams per kilogram (mg/kg) IV amphotericin B once daily and 25 mg/kg of 5-flucytosine every six hours via nasogastric tube. Head MRI and MRI total spine revealed chronic adhesive arachnoiditis (Figure 3).

Outcome and follow-up

Her CSF cultures cleared during hospitalization. Her seizures were medically controlled, and she was extubated. Amphotericin and flucytosine were discontinued after 2.5 weeks and oral fluconazole 800 mg/day was started, which continued until November 2021 per infectious disease service. She transitioned to acute care one month into her hospitalization as she was medically stable. She had another episode of decreased interaction with a stable head CT. Her exam returned to baseline, and she was later discharged to an acute rehab facility after 33 days of hospitalization at our facility. Her EVD was also weaned and removed prior to discharge. She had a CT scan of her head on the day of her discharge and was planned to have another CT after a month for follow-up, but she was later re-admitted due to worsening balance, incontinence, and increasing confusion. She underwent a VP shunt placement with improved functional status. Following her discharge, her symptoms improved. Her recovery was complicated by infection and cranial wound dehiscence with cultures growing methicillin-susceptible *Staphylococcus aureus* (MSSA), and her VP shunt had to be replaced on the other side. She continued to improve clinically following the replacement of her shunt. She was later discharged with her latest MRI and CT head imaging without evidence of additional infection and unchanged ventricular system.

## Discussion

Arachnoiditis is a relatively rare condition. The symptoms can present with non-specific neurological features such as back pain, radiation of pain down lower extremities, mobility restriction, and paraplegia, while it can also initially be asymptomatic [3,7,9]. It is a difficult diagnosis, and most are typically in the thoracic and cervical areas, although lumbosacral regions have been reported in the past [1-2,8,10]. The etiology of arachnoiditis is mainly infections or iatrogenic with injections, contrast, dyes, trauma, and other neurosurgical interventions [3,5,7]. For infectious etiologies, there are reports of tuberculosis, syphilis, cryptococcus, and candida tropicalis being causative species [4,6,9,11].

Furthermore, arachnoiditis can occur either as a primary lesion or following meningitis or tuberculosis of the spine [4,9,11]. Our patient has a known risk factor of subarachnoid hemorrhage and VP shunt placement (neurosurgical intervention). The source of infection was later found to be her VP shunt. Our patient initially presented with a history of subarachnoid hemorrhage and VP shunt placement with imaging findings consistent with arachnoiditis with abnormal CSF features.

Arachnoiditis can present with common MRI abnormalities such as arachnoid cysts, thickening, displacement, or clumping of nerve roots with contrast enhancement, cord swelling with T2 hyperintensity, arachnoid separation, cord compression, displacement tethering, or atrophy [8,10]. Delamarter et al. also developed a radiological staging of arachnoiditis with three groups. Group 1 showed adherent grouped nerve roots, group 2 with nerve roots that are adherent peripherally with an empty sac appearance, and group 3 with a mass replacing the subarachnoid space [10]. This patient’s symptoms would be consistent with Group 2 and 3 at the late imaging timepoint. Early on, her imaging matches groups 1 and 2 as some non-specific although abnormal features are seen with a possible grouping of roots and adherence to the periphery. Most interestingly, the lesion we saw on her imaging was away from her infectious site of origin, noting that it would have communicated through her CSF.

*Candida* infections are often difficult to diagnose in the setting of CNS infection [12-13]. Although MRIs can often detect an abscess, they can often be confused for tumors or other neoplastic disease conditions [12]. Furthermore, unremarkable CSF with only elevated protein is frequent in those abscesses and infections [12]. Our patient initially presented in the clinic as she had an unremarkable CSF study although without mass or meningeal enhancement concerning for abscess or meningitis. Although her CSF was unremarkable, our patient had abnormal CSF flow and imaging findings coupled with a high elevation in protein, which led us to suspect arachnoiditis and possible fungal infection. However, at this time, our patient was not symptomatic with infectious symptoms that we would have suspected *Candida* infection. Her only concerns were possible neurological symptoms of back pain that were resolved at the time, muscle spasms, and incontinence that could have resulted from her already chronic condition. Although she had the possible symptoms of arachnoiditis, they were relatively mild. They involved multiple spine levels, separated, and not initially having fevers, chills, or other infectious constitutional symptoms, which put doubt on the diagnosis of fungal infection and arachnoiditis.

*Candida* infections in medical devices and spinal abscesses are well described [12-17]. For shunts, infection occurs typically due to contamination at placement or hematogenous dissemination [13,14]. These fungal device infections are uncommon, with the estimated causative incidence being 1% of infections, but this may be underestimated due to culture-negative infections [14]. Furthermore, these abscesses can cause hydrocephalus and cord compression [12,15]. One case describes how *Candida albicans* can present with ventriculomegaly and, thus, hydrocephalus due to arachnoiditis as a cauda equina abscess [14]. However, for our patient, her infection initially presented as arachnoiditis due to most likely a VP shunt contamination and further led to dissemination with fever and shunt obstruction with worsening mental status. Delayed occurrence of spinal arachnoiditis has also been reported in the literature. Several case reports describe delay of symptoms following a neurosurgical intervention, with one case describing a seven-month delay in the occurrence of symptoms [15]. Our patient’s presentation from the initial infection was delayed as well. Her symptoms started with lower lumbar pain, then progressed to thoracic muscle spasms for multiple months, then acutely worsening to having infectious symptoms and imaging consistent with infectious origin. Furthermore, our patient later had another infection of her shunt with MSSA, which may be due to possible immune suppression as a result of her chronic infection but more likely due to repeated procedural burden as she had wound dehiscence, as she did not have any chronic conditions that may cause immunodeficiency. Thus, the progression of neurological symptoms and suspect imaging findings should be evaluated extensively, especially with the involvement of medical devices. Infectious seeding of medical devices should be considered in patients with arachnoiditis as their infectious course may be insidious, as with our patient.

## Conclusions

Clinical symptoms concerning arachnoiditis may benefit from performing MRI as part of the workup. Subtle and delayed progression of symptoms over months may be possible in arachnoiditis. If imaging demonstrates arachnoiditis, infectious etiologies, including fungal should be explored despite an indolent course. Although the patient may be asymptomatic at presentation, fungal infections should be considered, especially with recent device placement, even with normal CSF studies.
